# Neuroprotective Mechanisms of Porcine Brain Enzyme Hydrolysate in Memory Impairment: Multi-Target Strategy Against Amyloid-β-Induced Neurotoxicity

**DOI:** 10.3390/ijms26136030

**Published:** 2025-06-24

**Authors:** Sun Myung Yoon, Ye-Won Lee, Min Ju Kim, Jae-Joon Shin, Gun Won Bae, Sunmin Park

**Affiliations:** 1 Department of R&D, Unimed Pharmaceuticals Inc., Unimed Bldg., Seoul 05567, Republic of Korea; smyoon@unimed.co.kr (S.M.Y.); ywlee@unimed.co.kr (Y.-W.L.); alswn0512@unimed.co.kr (M.J.K.); sjj010@unimed.co.kr (J.-J.S.); gwbae33@unimed.co.kr (G.W.B.); 2 Department of Food and Nutrition, Obesity/Diabetes Research Center, Hoseo University, Asan-si 31499, Republic of Korea

**Keywords:** porcine brain enzyme hydrolysate, memory impairment, amyloid-β aggregation, cholinergic dysfunction, oxidative stress, neuroinflammation

## Abstract

This study investigated the potential neuroprotective mechanisms of porcine brain enzyme hydrolysate (PBEH) against Alzheimer’s disease pathology using differentiated SH-SY5Y cells. Differentiated neuronal cells were treated with 40 μM amyloid-β(1-42; Aβ) to induce neurotoxicity, followed by PBEH treatment (12.5–400 μg/mL), Com-A (peptide-based neuroprotective supplement; 200 μg/mL) treatment, and Com-B (herbal extract known for improving memory function; 100 μg/mL) treatment. Key assessments included cell viability, Aβ aggregation in adding 10 μM Aβ, amyloidogenic proteins (APP, BACE), synaptic markers (BDNF, ERK), apoptotic markers (BAX/BCL-2, caspase-3), oxidative stress (reactive oxygen species (ROS)), cholinergic function (ChAT, AChE), MAPK signaling (JNK, p38), and neuroinflammation (IL-1β). PBEH contained high concentrations of amino acids, including L-lysine (32.3 mg/g), L-leucine (42.4 mg/g), L-phenylalanine (30.0 mg/g) and the PSIS peptide (86.9 μg/g). Treatment up to 400 μg/mL showed no cytotoxicity and had cognitive protection effects up to 152% under Aβ stress (*p* < 0.05). PBEH significantly attenuated Aβ aggregation, decreased APP (28%) and BACE (51%) expression, enhanced synaptic function through increased BDNF, and restored ERK phosphorylation (*p* < 0.05). Anti-apoptotic effects included a 76% reduction in the BAX/BCL-2 ratio, a 47% decrease in caspase-3, and a 56% reduction in ROS levels. Cholinergic function showed restoration via increased ChAT activity (*p* < 0.01) and decreased AChE activity (*p* < 0.05). PBEH reduced IL-1β levels by 70% and suppressed JNK/p38 phosphorylation (*p* < 0.05). While Com-A enhanced BDNF and Com-B showed anti-inflammatory effects, PBEH demonstrated activity across multiple pathway markers. In conclusion, these findings suggest that PBEH may enable neuronal preservation through multi-pathway modulation, establishing foundational evidence for further mechanistic investigation in cognitive enhancement applications.

## 1. Introduction

Cognitive decline represents a critical early manifestation of neurodegenerative disorders such as Alzheimer’s disease (AD), characterized by progressive neuronal dysfunction linked to interconnected pathological mechanisms including cholinergic deficits, oxidative stress, neuroinflammation, synaptic degeneration, and impaired insulin signaling [1,2,3]. Current therapeutic approaches primarily focus on acetylcholinesterase (AChE) inhibitors, which demonstrate limited efficacy and are often constrained by adverse effects [1,4,5]. The complex interplay between multiple pathological pathways in memory dysfunction suggests that single-target approaches may be insufficient, highlighting the need for therapeutic strategies that can simultaneously modulate multiple disease mechanisms [6].

Natural bioactive compounds, particularly protein hydrolysates, have emerged as promising candidates for multi-target therapy [7]. Among these, porcine brain enzyme hydrolysate (PBEH) has shown potential advantages over existing neuroprotective compounds. N-pep-12 (Com-A) demonstrates neuronal protection effects primarily through targeted peptide-mediated mechanisms with established brain-derived neurotrophic factor (BDNF) enhancement properties [8]. *Ginkgo biloba* extract (Com-B) exerts its benefits mainly through antioxidant and anti-inflammatory activities documented in clinical studies, and it also is reported to improve memory function [9,10]. PBEH offers a multi-modal approach. PBEH is produced through the enzymatic hydrolysis of porcine brain tissue, yielding a complex mixture of bioactive peptides with potentially enhanced bioavailability, though their capacity to cross the intestinal barrier and blood–brain barrier (BBB) requires validation [11,12]. This diverse peptide composition may enable PBEH to simultaneously target multiple pathological pathways including cholinergic dysfunction, oxidative stress, neuroinflammation, and synaptic degeneration [13].

Despite promising preliminary observations, a critical knowledge gap exists: the precise cellular and molecular mechanisms underlying PBEH’s potential multi-modal effects remain poorly characterized compared to well-studied compounds like Com-A and Com-B. While the mechanisms of action for Com-A (N-pep-12) and Com-B (*Ginkgo biloba* water extract) are relatively well-defined through their established single-target approaches, PBEH’s complex multipeptide composition and hypothesized multi-target activity require detailed mechanistic investigation. This limitation hinders the optimization of PBEH’s therapeutic potential and impedes the development of more effective peptide-based interventions. Specifically, it remains unclear whether PBEH’s diverse bioactive peptides directly modulate cholinergic signaling, amyloid-β (Aβ) metabolism, oxidative stress responses, and synaptic plasticity at the cellular level or whether observed effects occur through indirect pathways or downstream signaling cascades.

To address this knowledge gap, we designed an *in vitro* study using differentiated SH-SY5Y cells, a well-established model for investigating neuronal function-related cellular processes [13,14,15]. This study aimed to characterize the efficacy of PBEH in modulating multiple pathological pathways involved in memory impairment, providing foundational insights for therapeutic development and identifying potential molecular targets for peptide-based interventions in neurodegenerative conditions.

## 2. Results

### 2.1. Amount of Index Compounds Present in PBEH and Cytotoxicity Assessment

PBEH was standardized with three amino acids including L-lysine (32.3 ± 0.66 mg/g), L-leucine (42.4 ± 0.36 mg/g), and L-phenylalanine (30.0 ± 0.44 mg/g) and one peptide, PSIS (86.9 ± 11.2 ug/g) (n = 3) (Supplementary Figure S1). No cytotoxicity was observed in the differentiated SH-SY5Y cells at PBEH concentrations of up to 400 μg/mL (Figure S2).

### 2.2. PBEH Reduction in Aβ Processing and Aggregation

**Figure 1:**
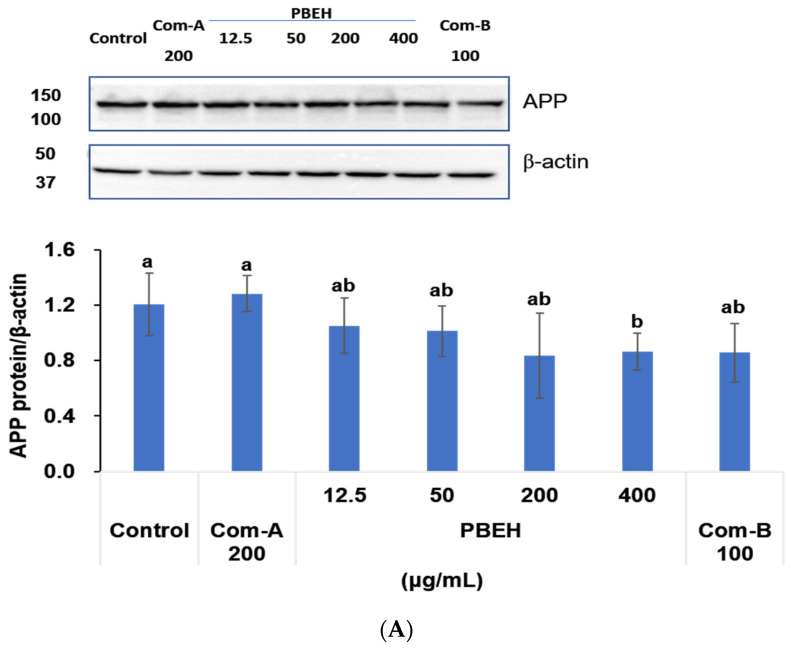

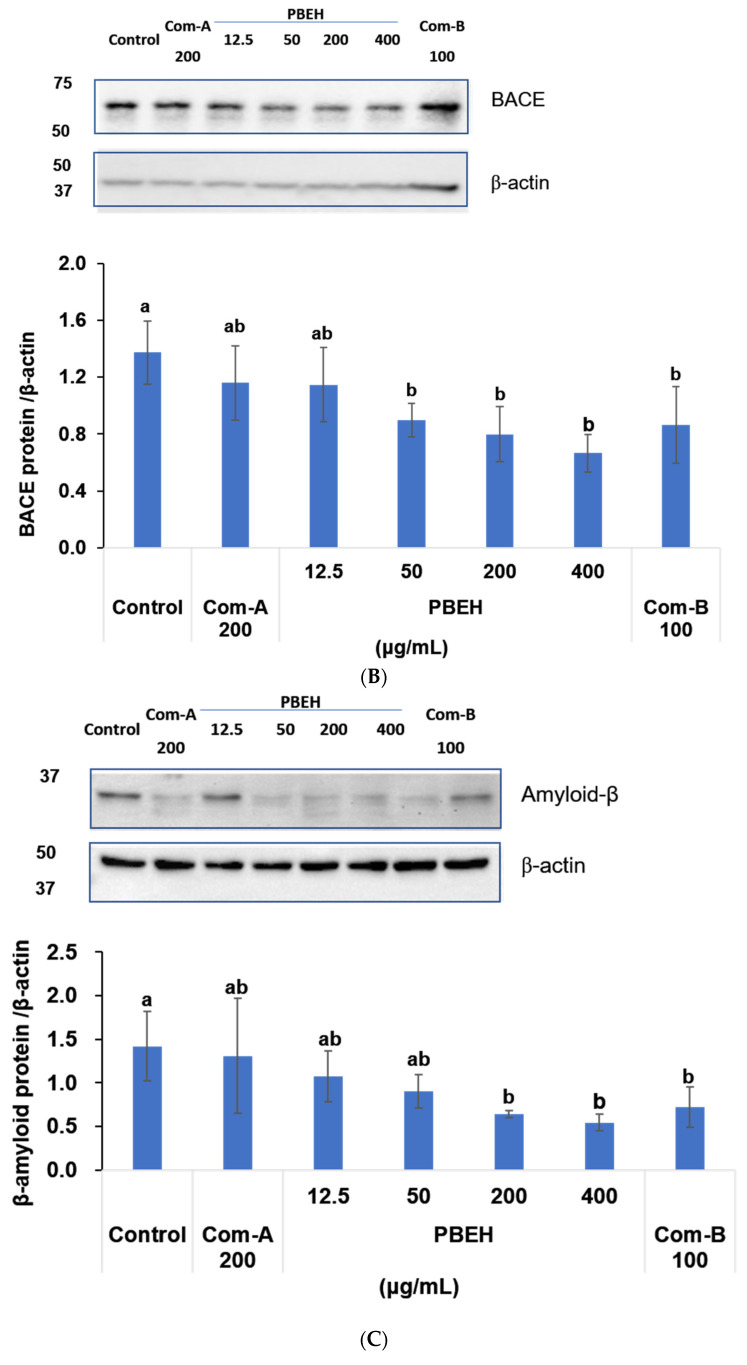

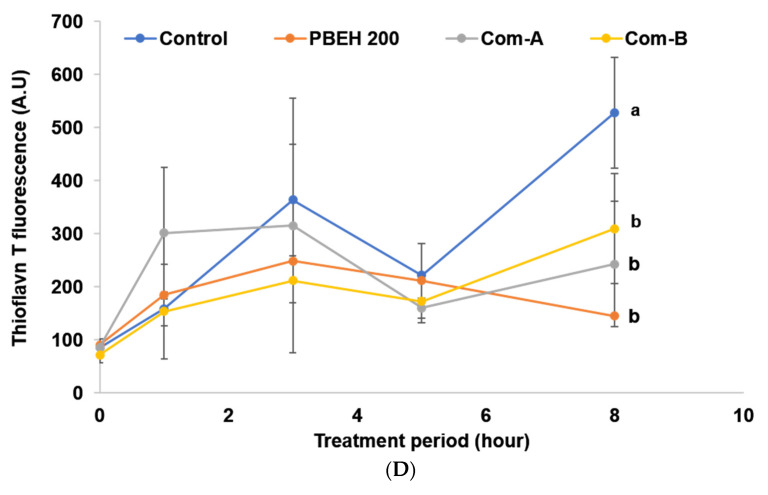
Effect of porcine brain enzyme hydrolysate (PBEH) on amyloid-β (Aβ) aggregation and amyloidogenic protein expression in Aβ-treated differentiated SH-SY5Y neuronal cells. (**A**) Amyloid precursor protein (APP) levels. (**B**) β-site APP cleaving enzyme (BACE) protein levels. (**C**) Amyloid-β (Aβ) protein levels. (**D**) Time course analysis of amyloid-β (Aβ) aggregation. Differentiated SH-SY5Y neuronal cells were treated with PBEH (200 μg/mL), Com-A (N-PEP-12), and Com-B (*Ginkgo biloba* extract), followed by exposure to 10 μM amyloid-β (Aβ). Amyloid-β (Aβ) aggregation was measured at 0, 2, 4, 6, and 8 h using thioflavin T fluorescence. Results are shown as means ± standard deviation (n = 3). a,b Different letters on bar or beside dot indicate significant differences among groups by Tukey’s test at *p* < 0.05 when one-way ANOVA showed statistically significant differences.

Aβ accumulation is directly linked to APP cleavage by β-site APP cleaving enzyme (BACE) and the subsequent aggregation in brain cells. In differentiated SH-SY5Y cells, Com-A (N-pep-12; 200 μg/mL) and Com-B (Ginkgo biloba leaf water extract; 100 μg/mL) served as positive controls for memory function [9,10], while PBEH was tested at concentrations of 12.5, 50, 200, and 400 μg/mL. PBEH treatment significantly reduced APP expression levels in a dose-dependent manner, with 400 μg/mL PBEH decreasing APP by 0.71-fold relative to the control (*p* < 0.05; Figure 1A). Com-B showed a tendency toward APP reduction but did not reach statistical significance. BACE protein expression was similarly suppressed by PBEH treatment, with 400 μg/mL PBEH reducing BACE levels by 0.49-fold compared to the control in a dose-dependent manner (*p* < 0.05; Figure 1B). Consistent with reduced amyloidogenic processing, Aβ protein levels were significantly lower in PBEH-treated groups compared to the control. PBEH at 200 and 400 μg/mL demonstrated superior inhibitory effects on Aβ levels compared to Com-B, with 400 μg/mL PBEH reducing Aβ by 0.38-fold compared to the control levels (*p* < 0.05; Figure 1C). Com-A treatment did not significantly modulate APP, BACE, or Aβ expression compared to the control.

To assess aggregation dynamics, differentiated SH-SY5Y cells were exposed to 10 μM Aβ(1-42) and monitored over 8 h. Progressive Aβ aggregation was observed in control cells throughout the observation period. While Com-A and Com-B treatments initially reduced aggregation rates until 5 h, PBEH demonstrated a sustained inhibition of Aβ aggregation throughout the entire 8 h period, showing the most consistent anti-aggregation effect (Figure 1D). PBEH (200 μg/mL) significantly reduced Aβ aggregation by 0.28-fold at 8 h compared to the control.

### 2.3. PBEH Attenuation of Oxidative Stress and Neuroinflammation

**Figure 2:**
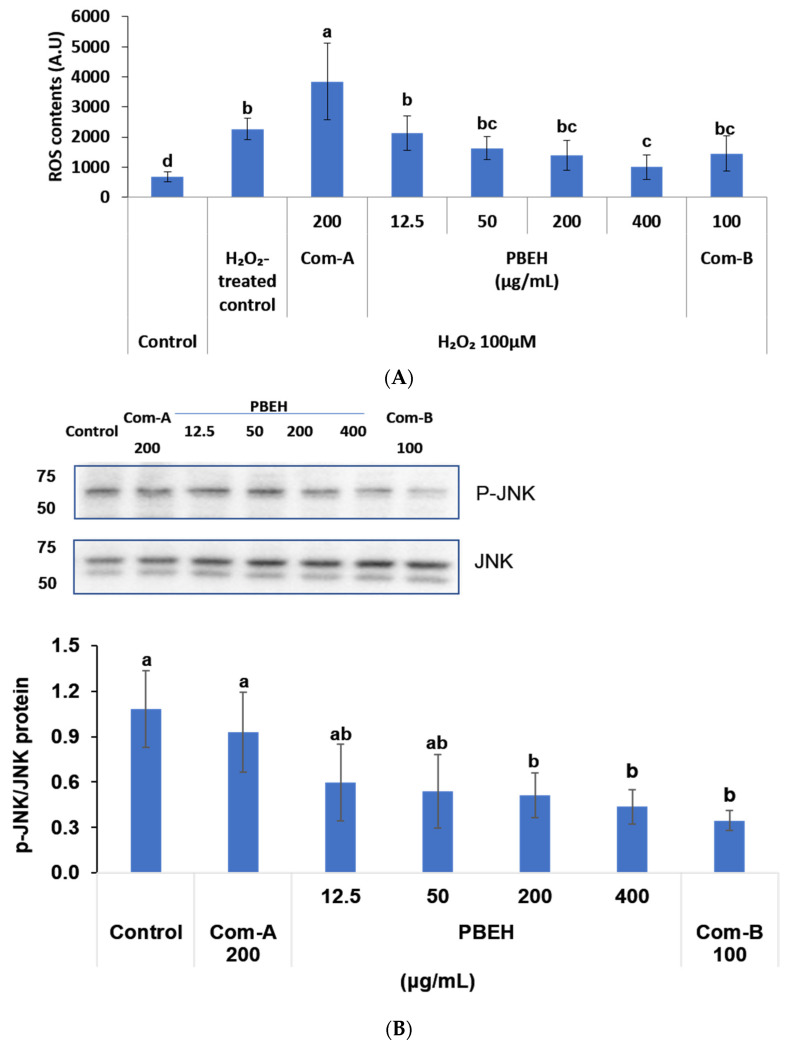

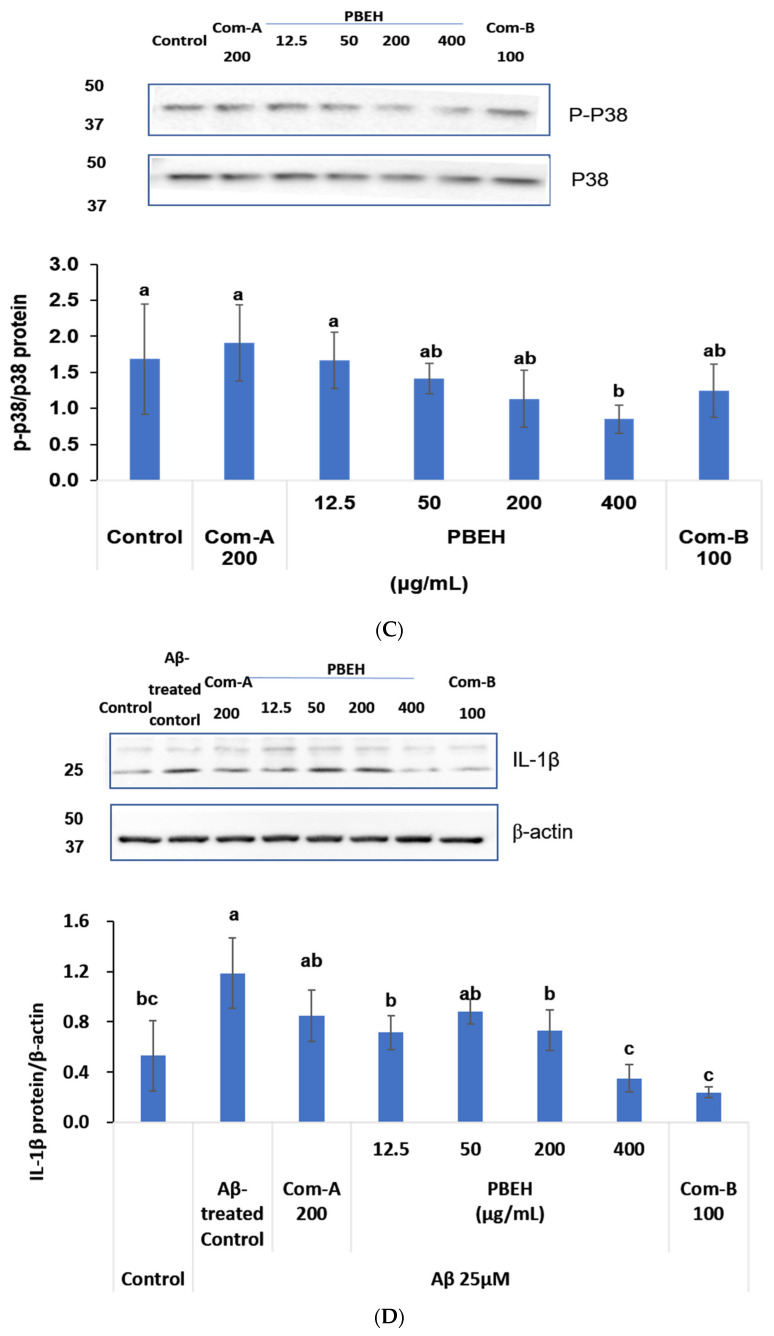
Effects of porcine brain enzyme hydrolysate (PBEH) on oxidative stress and neuroinflammation in differentiated SH-SY5Y neuronal cells. (**A**) Intracellular reactive oxygen species (ROS) levels in cell lysates after exposure to hydrogen peroxide (H_2_O_2_). (**B**) Ratio of phosphorylated c-Jun N-terminal kinase (p-JNK) to total JNK in cell lysates. (**C**) Ratio of phosphorylated p38 (p-p38) to total p38 in cell lysates. (**D**) Interleukin-1β (IL-1β) protein levels in supernatants of amyloid-β-(Aβ)-treated cells. Differentiated SH-SY5Y cells were treated with PBEH (12.5–400 μg/mL), Com-A (N-PEP-12), and Com-B (*Ginkgo biloba* extract). Results are shown as means ± standard deviation (n = 3). a,b,c Different letters on bar indicate significant differences among groups by Tukey’s test at *p* < 0.05 when one-way ANOVA showed statistically significant differences.

While Aβ aggregation generates reactive oxygen species (ROS) and neuroinflammation, they are bidirectionally interconnected, exacerbating the conditions. The administration of H_2_O_2_ (100 μM) substantially increased oxidative stress compared to untreated controls (Figure 2A). PBEH treatment demonstrated dose-dependent antioxidant effects, with 200 μg/mL and 400 μg/mL reducing ROS content by 39% and 56%, respectively, compared to H_2_O_2_-treated controls (*p* < 0.01; Figure 2A). Com-B (100 μg/mL) exhibited antioxidant efficacy comparable to PBEH at 200 μg/mL.

Elevated ROS levels trigger neuroinflammatory cascades, with JNK and p38 MAPK serving as key inflammatory mediators. In H_2_O_2_-treated differentiated SH-SY5Y cells, PBEH (400 μg/mL) significantly reduced JNK activation, decreasing the phosphorylated JNK-to-total JNK (p-JNK/JNK) ratio by 0.40-fold compared to the control (Figure 2B). Similarly, PBEH treatment substantially suppressed p38 MAPK phosphorylation, with 400 μg/mL PBEH reducing the phosphorylated p38-to-total p38 (p-p38/p38) ratio by 0.50-fold relative to the control (*p* < 0.05; Figure 2C). The p-p38/p38 ratio in PBEH-treated cells was restored to levels comparable to the Com-B-positive control group.

IL-1β, a key neuroinflammatory marker, was significantly elevated in Aβ-treated differentiated SH-SY5Y cells compared to normal controls without Aβ administration. PBEH treatment effectively suppressed this inflammatory response, with 400 μg/mL PBEH reducing IL-1β levels by 0.30-fold compared to Aβ-treated controls (*p* < 0.05; Figure 2D). This substantial reduction demonstrates PBEH’s ability to attenuate pro-inflammatory cytokine production in response to Aβ stimulation. These findings demonstrated that PBEH exerts comprehensive anti-inflammatory effects through multiple mechanisms: direct ROS scavenging, the suppression of pro-inflammatory MAPK signaling pathways (JNK and p38), and a reduction in inflammatory cytokine production.

### 2.4. PBEH Enhancement in Synaptic Plasticity Markers

**Figure 3:**
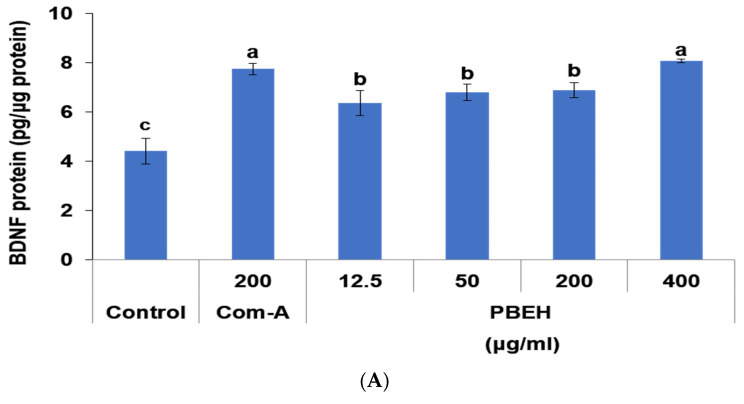

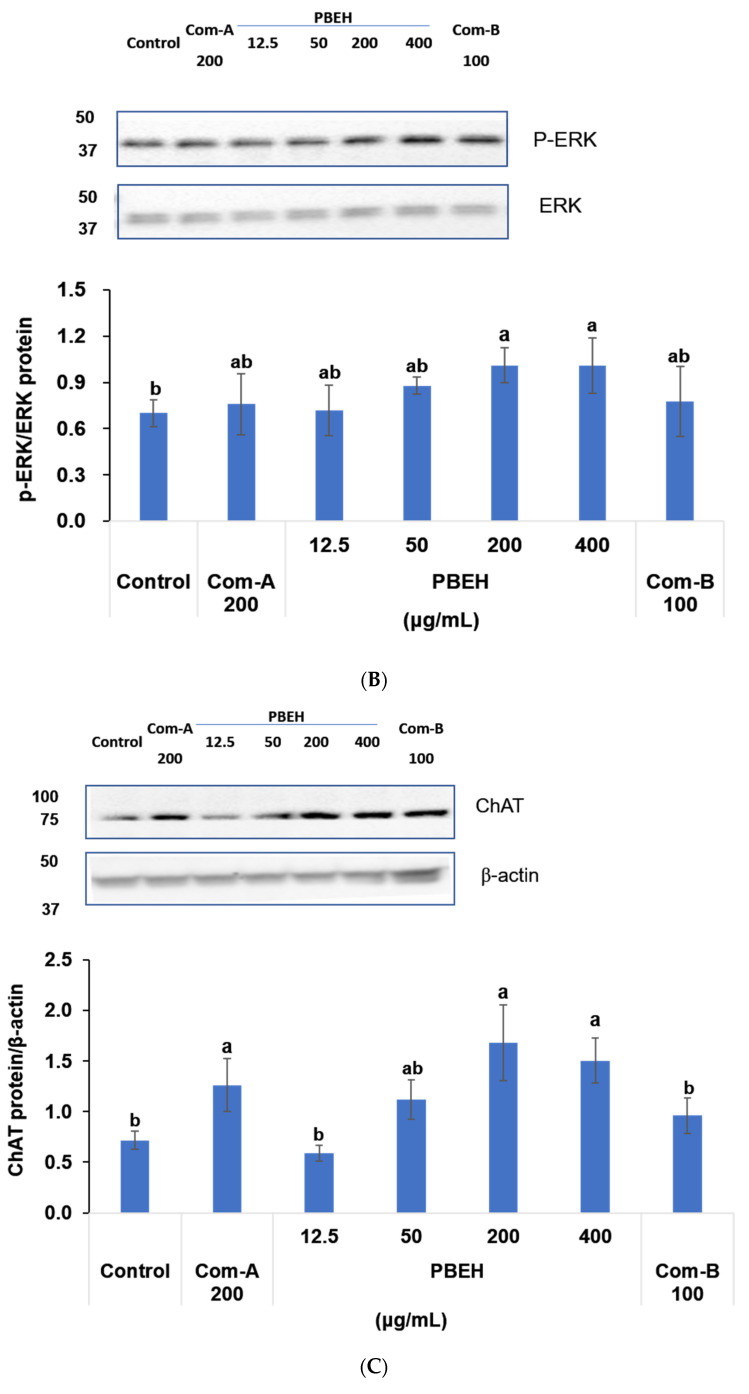

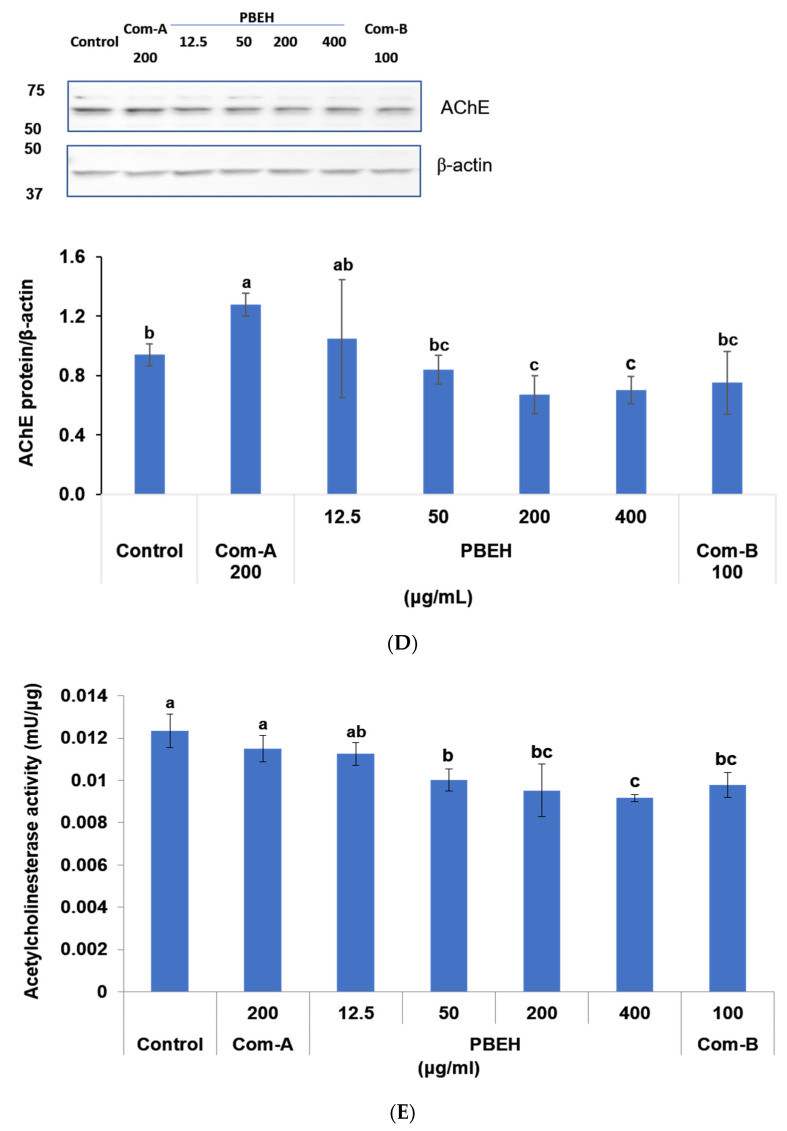
Effect of porcine brain enzyme hydrolysate (PBEH) on brain-derived neurotrophic factor (BDNF) expression and cholinergic markers in differentiated SH-SY5Y neuronal cells. (**A**) Brain-derived neurotrophic factor (BDNF) protein levels. (**B**) Ratio of phosphorylated extracellular signal-regulated kinase (p-ERK) to total ERK in cell lysates. (**C**) Choline acetyltransferase (ChAT) levels in cell lysates. (**D**) Acetylcholinesterase (AChE) protein expression levels in cell lysates. (**E**) Acetylcholinesterase (AChE) enzymatic activity in cell lysates. Results are shown as means ± standard deviation (n = 3). Differentiated SH-SY5Y cells were treated with PBEH (12.5–400 μg/mL), Com-A (N-PEP-12), and Com-B (*Ginkgo biloba* extract). a,b,c Different letters on bar indicate significant differences among groups by Tukey’s test at *p* < 0.05 when one-way ANOVA showed statistically significant differences.

BDNF serves as a critical protein for enhancing neuronal growth, survival, and synaptic plasticity. PBEH treatment significantly increased BDNF protein levels compared to the control, with 400 μg/mL PBEH enhancing BDNF expression by approximately 83% (*p* < 0.05; Figure 3A). Notably, the BDNF levels following PBEH (400 μg/mL) treatment were comparable to those achieved with Com-A (200 μg/mL), while both the levels after both treatments significantly exceeded control levels.

Synaptic plasticity is further regulated through ERK signaling pathways, which are involved in dendritic growth and synaptic consolidation. PBEH treatment (400 μg/mL) significantly increased the phosphorylated ERK-to-total ERK (p-ERK/ERK) ratio by 1.4-fold compared to the control (Figure 3B). This enhancement suggests that PBEH promotes synaptic plasticity and reduces synaptic degeneration through the activation of pro-survival ERK signaling.

### 2.5. PBEH Modulation of Cholinergic Function

Synaptic modulation indirectly affects acetylcholine content in synapses, thereby modulating cholinergic tone [16]. ChAT, the enzyme responsible for acetylcholine synthesis that stimulates neuronal activity to enhance memory function, was significantly increased following PBEH treatment. Both 200 and 400 μg/mL PBEH significantly elevated ChAT expression compared to the control, with 400 μg/mL PBEH increasing ChAT by 2.34-fold relative to the control (*p* < 0.01; Figure 3C). PBEH (200 μg/mL) elevated ChAT levels to those comparable with Com-A (200 μg/mL), though the levels achieved with Com-A treatment alone did not significantly differ from those of the control.

Conversely, the levels of AChE, which degrades acetylcholine, were reduced by PBEH treatment. PBEH (400 μg/mL) significantly decreased AChE protein expression by 25% compared to the control (*p* < 0.05; Figure 3D). Consistent with protein expression changes, AChE enzymatic activity was elevated in Aβ-treated control cells but was significantly reduced following PBEH treatment. PBEH (50–400 μg/mL) reduced AChE activity to levels comparable to Com-B (100 μg/mL), with approximately 25% reduction observed at the highest PBEH concentration (*p* < 0.05; Figure 3E). These findings demonstrate that PBEH enhances cholinergic function through a dual mechanism: increasing acetylcholine synthesis via ChAT upregulation while simultaneously suppressing acetylcholine degradation through a reduction in both AChE expression and enzymatic activity.

### 2.6. PBEH Anti-Apoptotic Activity in Neuronal Cells

**Figure 4:**
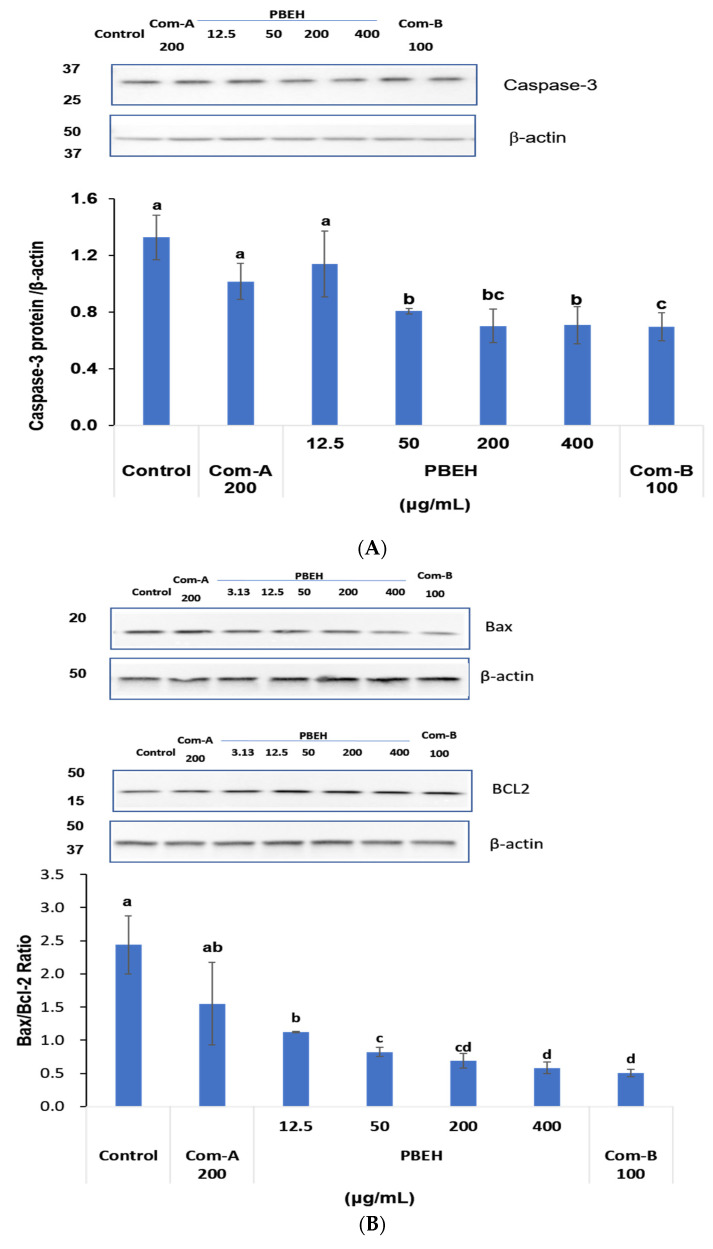

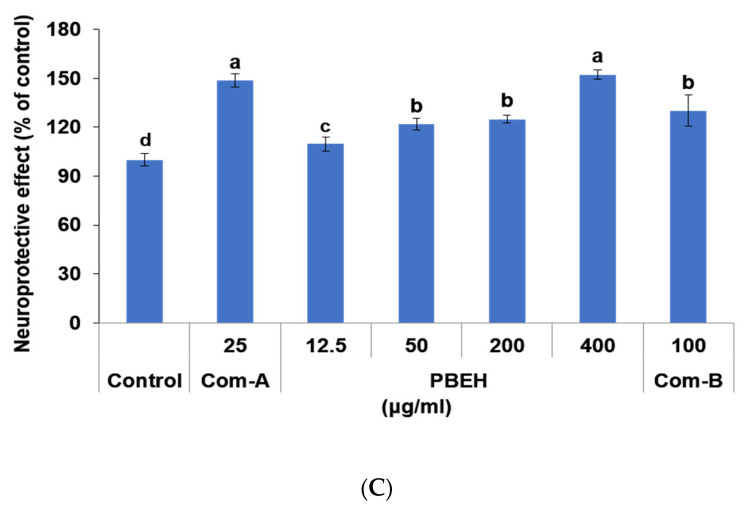
Effect of porcine brain enzyme hydrolysate (PBEH) on cell survival and apoptosis regulation in differentiated SH-SY5Y neuronal cells. (**A**) Caspase-3 protein levels in cell lysates. (**B**) Protein levels of apoptotic markers, including BCL2-associated X protein (BAX) and B-cell lymphoma 2 (BCL-2), as well as BAX/BCL-2 ratio. (**C**) Neuroprotective effect, as assessed by 3-(4,5-dimethylthiazol-2-yl)-2,5-diphenyltetrazolium bromide (MTT) assay. Results are shown as means ± standard deviation (n = 3). Differentiated SH-SY5Y cells were treated with PBEH (12.5–400 μg/mL), Com-A (N-PEP-12), and Com-B (*Ginkgo biloba* extract). a,b,c,d Different letters on bar indicate significant differences among groups by Tukey’s test at *p* < 0.05 when one-way ANOVA showed statistically significant differences.

Neuronal cell apoptosis was assessed through key apoptotic markers including caspase-3 protein levels and the BAX/BCL-2 ratio. PBEH treatment significantly reduced caspase-3 protein expression in a dose-dependent manner across concentrations of 50–400 μg/mL compared to the control (Figure 4A). The highest concentration of PBEH (400 μg/mL) achieved a 47% reduction in caspase-3 levels relative to the control, indicating the substantial suppression of the apoptotic execution pathway. Com-B (100 μg/mL) similarly demonstrated caspase-3 reduction, though to a lesser extent than PBEH treatment.

The BAX/BCL-2 ratio serves as a critical indicator of mitochondrial-mediated apoptotic status, where elevated ratios indicate pro-apoptotic conditions, and reduced ratios reflect anti-apoptotic, cell-protective states. PBEH treatment significantly modulated this apoptotic balance by suppressing pro-apoptotic BAX protein expression while maintaining anti-apoptotic BCL-2 levels. PBEH (400 μg/mL) dramatically reduced the BAX/BCL-2 ratio by 76% compared to the control (Figure 4B), indicating the robust inhibition of the intrinsic apoptotic pathway.

Consistent with the observed reductions in apoptotic markers, PBEH treatment enhanced overall neuronal cell viability. The MTT assay results demonstrated that PBEH (400 μg/mL) increased neuroprotective effects by approximately 1.52-fold compared to the control, confirming that the molecular changes in apoptotic proteins translate to improved cell survival under neurotoxic conditions. These findings collectively demonstrate that PBEH exerts potent anti-apoptotic effects through dual mechanisms: the suppression of pro-apoptotic signaling (reduced caspase-3 and BAX) and the maintenance of cell survival pathways preserved BCL-2, ultimately resulting in enhanced neuronal viability and protection against Aβ-induced neurotoxicity.

## 3. Discussion

The present study provides evidence that PBEH may offer neuronal protection by modulating multiple pathological pathways implicated in cognitive decline. Using differentiated SH-SY5Y cells, we established that PBEH showed no cytotoxicity at concentrations up to 400 μg/mL while demonstrating protective effects against Aβ-induced neurotoxicity. Table 1 summarizes the observed effects across key neurodegeneration pathways, revealing distinct activity profiles for each treatment. PBEH showed multi-pathway modulation across several markers, Com-A exhibited particularly strong synaptic plasticity enhancement (BDNF), while Com-B demonstrated pronounced anti-apoptotic and anti-inflammatory responses. The magnitude of PBEH’s effects was comparable to Com-B across several cognitive function-related parameters, though direct mechanistic comparisons require further validation. These preliminary findings suggest that PBEH’s multi-target activity pattern warrants investigation as a potential candidate for conditions characterized by memory function impairment.

Aβ accumulation represents a core pathological feature in various neurodegenerative disorders linked to memory impairment. Aβ peptides are produced through the sequential cleavage of APP by BACE and γ-secretase [17]. Once generated, these peptides can aggregate to form oligomers and fibrils that disrupt synaptic function, trigger oxidative stress, and ultimately lead to neuronal death—a cascade of events central to memory dysfunction [18]. Our findings indicate that PBEH exerts protective effects against Aβ toxicity through multiple mechanisms. PBEH significantly reduced Aβ aggregation by approximately 72.5% at 8 h post-treatment, demonstrating superior efficacy to Com-B. Ab aggregation is related to a dose-dependent decrease in APP and BACE protein contents, thus targeting the upstream mechanisms of Aβ generation. Compared to Com-B as a positive control, it demonstrated similar effects as PBEH on APP and BACE levels and reduced Aβ aggregation by 53% at these same time points, suggesting time-dependent variations in efficacy between these compounds. Previous studies have shown that Com-B inhibits Aβ fibril formation through interactions with aromatic residues in the Aβ sequence, disrupting β-sheet formation [19,20]. Similarly, Com-A has been reported to reduce Aβ deposition in animal models of Alzheimer’s disease induced by D-galactose and aluminum chloride (AlCl_3_) [21]. This multi-targeted approach to Aβ pathology is particularly noteworthy, as PBEH not only reduces existing Aβ aggregation but also inhibits the production of new Aβ peptides. Such dual action may provide more comprehensive protection against Aβ-mediated neurotoxicity compared to agents that target only a single aspect of Aβ pathology.

Oxidative stress and apoptotic neuronal death represent interconnected pathological processes in neurodegenerative disorders [3]. Excessive ROS triggers mitochondrial dysfunction which disrupts the balance between pro-apoptotic and anti-apoptotic proteins and ultimately activates apoptotic cascades that lead to neuronal loss and memory impairment [22]. Our results demonstrate that PBEH exhibits significant anti-apoptotic properties by suppressing pro-apoptotic BAX while enhancing anti-apoptotic BCL-2 expression, resulting in a decreased BAX/BCL-2 ratio and a dose-dependent reduction in caspase-3 levels (50–400 μg/mL). Similarly, treatment with Com-B resulted in a reduced BAX/BCL-2 ratio and caspase-3 level. A previous study in post-stroke mice demonstrated that Com-B exerts its anti-apoptotic effects on hippocampal neurons by regulating the expression of BAX/BCL-2 and caspase-3 [23]. Additionally, PBEH demonstrated potent antioxidant capacity, reducing H_2_O_2_-induced ROS levels by up to 56% at 400 μg/mL. Com-B (100 μg/mL) exhibited antioxidant efficacy comparable to PBEH (200 μg/mL), consistent with its well-documented antioxidant properties [24]. Although direct comparisons between these compounds have been limited, the superior anti-apoptotic and antioxidant efficacy of PBEH represents a significant advantage in neuroprotective activity against oxidative stress, which is a trigger and consequence of Aβ pathology.

Beyond its protective actions against neurotoxic insults, PBEH also demonstrated neurotrophic properties by enhancing BDNF expression, a key protein for memory function in the brain [25]. Furthermore, PBEH increased the p-ERK/ERK ratio. This effect was observed at concentrations as low as 12.5 μg/mL, suggesting that PBEH may promote neuronal survival and synapse plasticity even at relatively low doses. Com-A showed significant increases in BDNF levels, comparable to PBEH at higher concentrations. Previous studies have reported that Com-B enhances BDNF expression through activating cAMP response element-binding protein (CREB) [26], which may explain its efficacy in our study. The ability to increase BDNF levels is particularly relevant for memory function, as BDNF plays a crucial role in synaptic plasticity and long-term potentiation—processes fundamental to learning and memory formation [27]. This aspect of PBEH’s mechanism represents a significant advantage over compounds that primarily target pathological processes without supporting regenerative pathways.

Acetylcholine is a critical neurotransmitter that modulates memory function by regulating synaptic plasticity and BDNF expression in the hippocampus and cortex, regions essential for learning and memory formation [28]. Our findings also reveal that PBEH positively modulates cholinergic function through dual mechanisms: increasing ChAT protein content while simultaneously suppressing both AChE protein expression and enzymatic activity. Com-A significantly increased ChAT levels but showed no significant inhibitory effect on AChE activity, suggesting a selective effect on acetylcholine synthesis rather than degradation. Conversely, Com-B exhibited a significant inhibition of AChE activity and protein expression but showed less pronounced effects on ChAT compared to PBEH at higher concentrations. Previous studies have established Com-B’s AChE inhibitory properties [29,30], but comprehensive evaluations of its effects on the cholinergic system, particularly in direct comparison with other compounds like PBEH, have been limited. This balanced modulation of the cholinergic system by PBEH probably results in enhanced acetylcholine levels, which are essential for proper cognitive function and memory formation. The ability of PBEH to enhance cholinergic transmission through multiple targets provides an advantage over conventional health supplements like Com-B, which often focus on a single aspect of cholinergic function.

Neuroinflammation is increasingly recognized as a critical contributor to memory impairment and neurodegenerative processes [31]. Our study demonstrated that PBEH reduced the production of IL-1β, a pro-inflammatory cytokine, in differentiated SH-SY5Y neuronal cells. Further investigation into the underlying mechanisms revealed that PBEH modulates the MAPK signaling pathways in a neuroprotective manner. Specifically, PBEH reduced the elevated p-JNK/JNK and p-p38/p38 ratios. PBEH inhibited stress- and inflammation-related signaling (JNK and p38 pathways). Com-B exhibited similar effects on the MAPK pathways, consistent with previous studies documenting its anti-inflammatory properties [32]. However, our data on these pathways of Com-A were less pronounced, particularly those about JNK and p38 inhibition, suggesting potential differences in the anti-inflammatory mechanisms between these compounds. Such comprehensive regulation of MAPK signaling probably contributes to the overall neuroprotective and anti-inflammatory effects of PBEH. Previous research has identified various botanical compounds that modulate the MAPK pathways to improve memory function [33]. However, the specific pattern of MAPK regulation by PBEH observed in our study represents a novel finding with important implications for understanding its mechanism of action.

**Figure 5:**
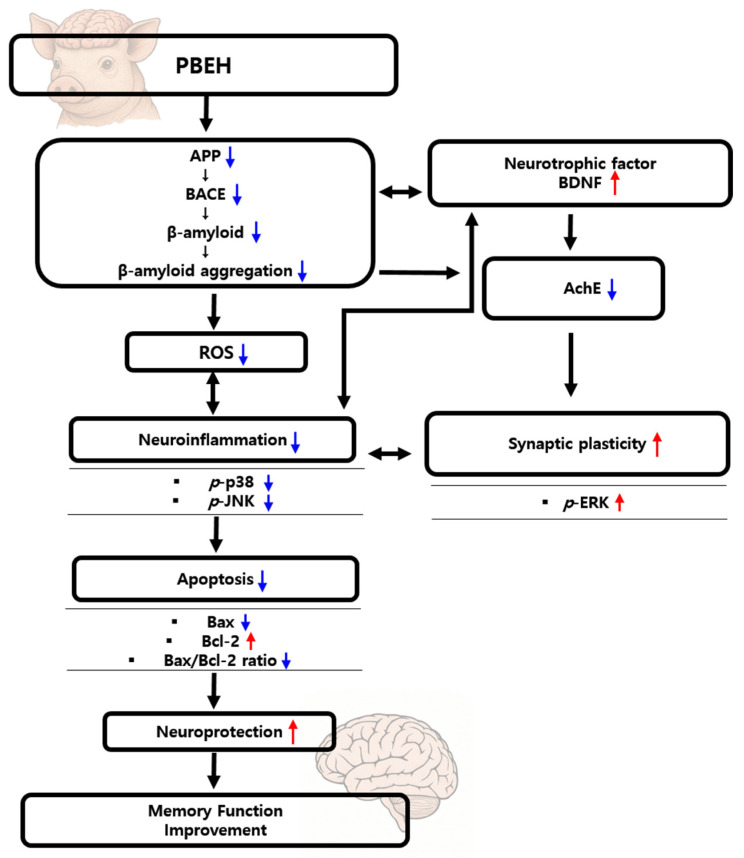
A proposed mechanistic model of porcine brain enzyme hydrolysate (PBEH)-mediated improvement in memory and cognitive function in neuronal cells. The diagram illustrates the multi-target effects of PBEH on key pathological pathways linked to Alzheimer’s disease. Amyloid-β (Aβ) aggregation, initiated by the increased expression of amyloid precursor protein (APP) and β-site APP cleaving enzyme (BACE), leads to neuroinflammation, oxidative stress, reduced synaptic plasticity, and impaired cognitive function. PBEH treatment downregulates APP and BACE expression, thereby reducing Aβ accumulation and aggregation. Additionally, PBEH attenuates neuroinflammation and oxidative stress while upregulating brain-derived neurotrophic factor (BDNF) and enhancing choline acetyltransferase (ChAT) activity, collectively promoting synaptic repair and cholinergic neurotransmission. In parallel, PBEH inhibits acetylcholinesterase (AChE), further enhancing cholinergic function. These neuroprotective effects are accompanied by the modulation of mitogen-activated protein kinase (MAPK) signaling pathways, including the suppression of phosphorylated p38 and c-Jun N-terminal kinase (JNK) and the activation of extracellular signal-regulated kinase (ERK), all contributing to improved synaptic plasticity and neuronal survival. Together, these coordinated actions lead to enhanced memory and cognitive function. The red and blue arrows indicate increases and decreases, respectively.

The present study evaluated the potential neuronal protective and cognitive enhancement effects of PBEH in an *in vitro* model of Alzheimer’s disease, wherein neuronal cells were challenged with Aβ peptides to mimic Aβ-induced neurotoxicity and synaptic dysfunction. As shown in Figure 5, Aβ exposure led to significant Aβ aggregation, increased neuroinflammation, and suppressed synaptic plasticity—memory function decline. Treatment with PBEH attenuated Aβ aggregation, potentially through the modulation of amyloidogenic pathway components such as APP and BACE, which may limit the accumulation of toxic Aβ species. PBEH treatment also correlated with reduced neuroinflammation, as evidenced by suppressed inflammation-related signaling pathways. This anti-inflammatory response was accompanied by increased BDNF levels, which may support synaptic remodeling while potentially counteracting inflammatory processes, suggesting a dual protective mechanism that warrants further mechanistic validation.

Moreover, PBEH inhibited AChE activity, leading to enhanced cholinergic neurotransmission, an essential process for memory formation. In parallel, PBEH modulated key components of the MAPK signaling cascade by downregulating stress kinases p38 and JNK while upregulating ERK, which is implicated in long-term potentiation and memory consolidation. However, Com-A demonstrated that an increase in BDNF content showed minimal impact on AChE activity or MAPK rebalancing. Com-B, on the other hand, exhibited moderate anti-inflammatory and antioxidant effects and mildly improved synaptic plasticity but did not exert a strong influence on Aβ processing or cholinergic signaling in our model. In contrast, PBEH exerted a more integrated effect across multiple interconnected pathways, targeting amyloid burden, neuroinflammation, synaptic regulation, cholinergic transmission, and intracellular signaling, culminating in a more robust restoration of memory and cognitive function.

While our *in vitro* findings are promising, several limitations should be acknowledged. First, the SH-SY5Y cell model, being an immortalized, non-primary, tumor-derived cell line [34], does not fully represent the complexity of primary neurons or the human brain’s multifactorial nature of memory impairment in vivo. The simplified cellular environment may not reflect the intricate neural networks and glial interactions present in intact brain tissue. Second, regarding bioavailability, while PBEH contains potentially bioactive compounds like leucine, L-phenylalanine, and the peptide PSIS that may undergo intestinal absorption, their capacity to traverse the blood–brain barrier remains uncertain, particularly for larger peptide components. Future studies should employ BBB permeability assessment models such as parallel artificial membrane permeability assay (PAMPA-BBB) to evaluate brain penetration potential, combined with the LC-MS tracking of specific bioactive peptides in plasma and cerebrospinal fluid to confirm central nervous system bioavailability. Finally, this study provided correlative evidence rather than establishing direct molecular mechanisms. Our findings suggest the effects of PBEH treatment and the modulation of various neuronal markers, but we did not demonstrate direct binding interactions with target proteins such as APP or BACE1. The observed effects on amyloidogenic pathways may result from direct enzyme inhibition, indirect regulatory mechanisms, or downstream signaling cascade modulation. Future mechanistic validation studies are essential to establish causative relationships and elucidate the precise molecular targets of PBEH components. These should include molecular binding assays, enzyme kinetic analyses, receptor interaction studies, and targeted inhibitor/knockdown experiments to determine the specific mechanisms underlying the observed neuroprotective effects.

The identification of specific bioactive constituents responsible for PBEH’s neuronal protective effects remains a critical knowledge gap. Comprehensive structure–activity relationship investigations, molecular docking analyses, enzyme kinetic characterizations, and binding affinity measurements would facilitate the identification of key therapeutic components. Such mechanistic characterization would enable direct comparisons with established active compounds in Com-B, including ginkgolides, bilobalide, and flavonoid constituents, potentially revealing synergistic or complementary mechanisms of action. Furthermore, translational studies addressing pharmacokinetic properties, including bioavailability profiles, blood–brain barrier penetration kinetics, and optimal dosing regimens, are prerequisites for clinical development. Comprehensive safety assessments, including long-term toxicology studies, would be essential before advancing to human trials. These foundational studies would transform our current observational findings into mechanistically grounded therapeutic applications for memory function disorders.

## 4. Materials and Methods

### 4.1. Preparation of PBEH and Measurement of Its Primary Components as Index Compounds

The porcine brain was perfused with distilled water to remove blood, and ethanol was added to dissolve lipid components and precipitate proteins. The protein precipitates were digested with protease at 40 °C for 20 h, then heat-inactivated. Undigested proteins were precipitated with ethanol and removed by microfiltration. The resulting hydrolysate was concentrated by evaporation, sterilized, and freeze-dried to yield PBEH powder. The PBEH powder contained various amino acids and peptides with molecular weights ranging from 2 to 5 kDa. Among these components, three amino acids (L-lysine, L-leucine, and L-phenylalanine) and one tetrapeptide (PSIS; proline–serine–isoleucine–serine) were utilized as index components to standardize the PBEH manufacturing process [35].

The amino acid content in PBEH was analyzed using an amino acid analyzer (LA8080, Hitachi, Chiyoda, Tokyo, Japan). Standard stock solutions containing the target amino acids were serially diluted with sample dilution buffer to prepare calibration standards at final concentrations of 1, 5, 25, and 75 mg/mL, then filtered through 0.45 μm membranes. Test samples were diluted 4000-fold with distilled water and similarly filtered before analysis. Amino acids were separated using a 100 μL injection volume with sodium citrate buffers, regeneration solution, and ninhydrin reagent. Detection was performed at the 570 nm and 440 nm wavelengths, with a reaction temperature of 130 °C and a flow rate of 0.45 mL/min. Quantification was achieved by comparing the peak areas of test solutions with standard curves based on retention times.

PSIS was quantified using ultra-performance liquid chromatography–mass spectrometry (UPLC-MS; Xevo TQ-XS, Waters, Milford, MA, USA). Standard stock solutions were prepared by dissolving PSIS (Anigen Co., Gwangju, Republic of Korea) in distilled water at 10 mg/mL and serially diluting it to five concentrations for calibration. Test solutions were prepared by diluting PBEH 1000-fold with distilled water and filtering through 0.45 μm membranes. Chromatographic separation was performed using a mobile phase of water/formic acid (99.9:0.1, *v*/*v*) and acetonitrile/formic acid (99.9:0.1, *v*/*v*), with a flow rate of 0.2 mL/min and column temperature of 30 °C. Detection employed electrospray ionization in positive mode (ESI+) with triple quadrupole mass spectrometry, monitoring the *m*/*z* 403.2 ion of PSIS. Quantification was based on peak areas in extracted ion chromatograms compared to calibration standards.

### 4.2. Cell Culture and Treatment Conditions

SH-SY5Y neuronal cells (Korea Cell Line Bank, Seoul, Korea) were cultured in Minimum Essential Medium (MEM) Media 1640 (Cytiva, Marlborough, MA, USA) supplemented with 10% fetal bovine serum (FBS; Hyclone, Marlborough, MA, USA) and 1% penicillin–streptomycin (Cytiva). Cells were maintained in 75 cm^2^ flasks at 37 °C under 5% CO_2_. When cell confluence reached 70%, cells were collected after enzymatic dissociation using trypsin–ethylenediamine tetraacetic acid (EDTA), resuspended in a complete medium, and plated in appropriate well plates. Once cells reached approximately 50–60% confluency, neuronal differentiation was induced by treating cells with 10 μM all-trans retinoic acid (Sigma-Aldrich, St. Louis, MO, USA) in DMEM containing 10% FBS for five days.

All experiments were performed in a blinded manner with coded treatments, and identities were revealed only after data analysis completion. For Western blot analysis, samples were loaded in a predetermined order (Control, Com-A, PBEH 12.5, 50, 200, 400 μg/mL, Com-B) due to technical requirements, but blinding was maintained through position-based coding during quantification and analysis.

### 4.3. Positive Control Compounds

Two positive control compounds were used for comprehensive mechanistic comparison with PBEH treatment. Com-A (N-PEP-12, 200 μg/mL) is a peptide-based supplement derived from hydrolyzed porcine brain proteins that have been demonstrated to enhance cognitive performance and neuronal plasticity in aged animal models [36,37]. Com-A exerts its neuroprotective effects primarily through the upregulation of BDNF and the modulation of synaptic plasticity pathways, including CREB and ERK signaling cascades [38,39]. Com-B consists of *Ginkgo biloba* leaf water extract (100 μg/mL; Pharmapia, Seoul, Korea) and is widely recognized for its neuroprotective and cognitive-enhancing properties, including improved cerebral blood flow, antioxidative effects, and neurotransmitter modulation in various in vivo and *in vitro* models [40]. Com-B also demonstrates antioxidant activity through ROS reduction, the suppression of pro-inflammatory cytokines (including IL-1β), the inhibition of AChE, and the modulation of MAPK pathways including p38 and JNK [32,41]. Both positive control compounds were selected to provide mechanistic comparisons with PBEH across the multiple pathological pathways investigated in this study.

### 4.4. Treatment Protocol

Differentiated cells were treated with PBEH at concentrations of 12.5, 50, 200, and 400 μg/mL to evaluate its neuroprotective effects and mechanisms in memory impairment models. Control groups received the solvent used to dissolve PBEH or positive control compounds. Normal control groups without Aβ or H_2_O_2_ inducers were included for baseline comparison.

### 4.5. Evaluation of Cytotoxicity

Differentiated SH-SY5Y neuronal cells were seeded into 24-well plates at a density of 2 × 10^5^ cells per well. After 24 h, the cells were treated with PBEH at concentrations of 12.5, 50, 200, and 400 µg/mL and incubated for an additional 48 h at 37 °C under 5% CO_2_. After treatment, the cells were washed with PBS and incubated with 500 μL of 3-(4,5-dimethylthiazol-2-yl)-2,5-diphenyltetrazolium bromide (MTT solution; 0.5 mg/mL) for 2–3 h. The medium was discarded, and the resulting formazan crystals were solubilized in dimethyl sulfoxide (DMSO). Absorbance was measured at 570 nm using a microplate reader to assess cell viability.

### 4.6. Evaluation of Neuroprotective Effects of PBEH

The differentiated SH-SY5Y neuronal cells in a 24-well plate at a density of 2 × 10^5^ cells per well were treated with PBEH at concentrations of 12.5, 50, 200, or 400 μg/mL for 24 h, followed by stimulation with Aβ(1-42) (Bachem, Bubendorf, Switzerland), as a neurotoxic stimulus, at final concentrations of 40 μM for an additional 24 h. Cell viability was determined using an MTT assay.

### 4.7. Aβ Aggregation Measured by Thioflavin T Fluorescence Analysis

The differentiated SH-SY5Y neuronal cells in a 24-well plate at a density of 3 × 10^5^ cells per well were then treated with solvent control, PBEH (200 μg/mL), Com-A (200 μg/mL), or Com-B (100 μg/mL) for 24 h, followed by exposure to Aβ(1-42) (10 μM) for an additional 24 h. After treatment, cells were washed with PBS and incubated at 37 °C in PBS containing thioflavin T for 8 h. Fluorescence measurements were taken at 0, 1, 3, 5, and 8 h using excitation and emission wavelengths of 450 and 482 nm, respectively. The data represented the inhibition rate (%) of each sample compared to the solvent control, which was set as 100% at each time point.

### 4.8. Evaluation of Production of Neurotrophic Factors

The differentiated SH-SY5Y neuronal cells were seeded in a 6-well plate at a density of 2 × 10^5^ cells per well and were treated with PBEH for 24 h. The cells were lysed with radio-immunoprecipitation assay (RIPA) buffer, and the lysates were collected to measure the levels of neurotropic factor, the BDNF, using the Human BDNF Picokine ELISA Kit (EK0307; Boster Bio, Pleasanton, CA, USA) according to the manufacturer’s protocol. At the end of the ELISA, absorbance was measured at 450 nm within 30 min in the Microreader. The amount of amyloid precursor protein (APP) and A in the cell lysates was measured using a Western blot assay.

### 4.9. Assessment of Acetylcholinesterase (AChE) Inhibition by PBEH

The inhibitory effect of PBEH on AChE activity was measured in the differentiated SH-SY5Y neuronal cells. The cells were seeded in a 6-well plate at a density of 2 × 10^5^ cells per well and treated with different amounts of PBEH for 48 h. Cell lysates were collected and centrifuged at 1500 rpm for 5 min, and the supernatants were used for AChE activity analysis using the Acetylcholinesterase Assay Kit (Abcam, Waltham, MA; ab138871) according to the manufacturer’s protocol. At the end of the assay, absorbance was measured at 410 nm, and AChE inhibitory activity was determined. The levels of choline acetyltransferase (ChAT), the enzyme that catalyzes the biosynthesis of acetylcholine, were measured in the lysate using a Western blot assay.

### 4.10. Measurement of Reactive Oxygen Species (ROS) Scavenging

The differentiated SH-SY5Y neuronal cells were seeded at a density of 8 × 10^4^ cells per well in a 48-well plate and treated with PBEH the following day. After 24 h, the cells were exposed to 100 μM hydrogen peroxide (H_2_O_2_) for an additional 24 h. The cells were then washed with PBS and incubated with 20 μM 2′,7′-dichlorodihydrofluorescein diacetate (DCF-DA) in PBS at 37 °C for 30 min. The fluorescence intensity was measured using excitation and emission wavelengths of 495 nm and 515 nm, respectively, to evaluate intracellular ROS generation.

### 4.11. Western Blot Analysis

Differentiated SH-SY5Y neuronal cells were seeded at a density of 2 × 10^5^ cells per well in a 6-well plate and treated with PBEH for 24 h. After washing with PBS, the cells were lysed with RIPA buffer, and the lysates were centrifuged at 12,000 rpm for 10 min to collect the supernatants. Protein concentrations were measured using the Lowry assay, and 20–30 μg of protein was loaded onto a 10% sodium dodecyl sulfate–polyacrylamide gel electrophoresis (SDS-PAGE) gel. After electrophoresis at 70 V, the proteins were transferred to a polyvinylidene fluoride membrane (PVDF) using wet transfer (0.4 A for 90 min). The membrane was blocked with 5% bovine serum albumin (BSA) for 1 h and incubated overnight at 4 °C with primary antibodies (1:1000 dilution) against designated primary antibodies as follows: Beta-amyloid B-4 (Santa Cruz, Dallas, TX, USA; sc-28365), BCL-2 (Cell Signaling, Danvers, MA, USA; 3498S), BCL2-associated X, apoptosis regulator BAX (Cell Signaling; 89477S), AChE (Genetex, Irvine, CA, USA; GTX101648), choline acetyltransferase, (ChAT, Abcam; Cambridge, UK; ab181023), BACE (Cell Signaling; 5606S), p44/42 mitogen-activated protein kinase (MAPK, Erk1/2) (Cell Signaling, 9102S), phospho-p44/42 MAPK (Erk1/2) (Cell Signaling, 9101S), Stress-Activated Protein Kinase/c-Jun N-terminal kinase (SAPK/JNK, Cell Signaling, 9252S), Phospho-SAPK/JNK (Thr183/Tyr185) (Cell Signaling, 9251S), p38 MAPK (Cell Signaling, 9212S), Phospho-p38 MAPK (Thr180/Tyr182) (Cell Signaling, 9211S), caspase-3 (Cell signaling; 9662), interleukin-1 beta (IL-1β; Cell Singling, 112242S), and β-Actin (Santa Cruz, sc-47778).

To assess IL-1β expression, cells were co-treated with PBEH and Aβ for 24 h, and lysates were prepared accordingly. The membrane was washed three times with Tris-buffered saline with Tween 20 (TBST, 0.1% Tween-20) and incubated with horseradish peroxidase (HRP)-conjugated secondary antibodies (Cell Signaling; 7074S for rabbit IgG, 7076S for mouse Immunoglobulin G [IgG]) for 1 h at room temperature. Protein bands were detected using enhanced chemiluminescence (ECL) reagents and analyzed for expression levels. Protein bands were quantified using ImageJ software (version 1.54g).

### 4.12. Statistical Analysis

All experiments were performed in triplicate, and data were presented as the mean ± standard deviation (SD). Statistical significance was determined using a one-way ANOVA followed by Tukey’s post hoc test, with a *p*-value < 0.05 considered statistically significant.

## 5. Conclusions

PBEH may offer neuronal protection against multiple pathological processes implicated in cognitive decline. A systematic comparison with established control compounds revealed that PBEH showed activity patterns comparable to Com-B across six key pathways: (1) associations with reduced Aβ production and aggregation through the modulation of APP and BACE levels; (2) anti-apoptotic responses indicated by altered BAX/BCL-2 ratios and caspase-3 levels; (3) oxidative stress mitigation through enhanced ROS scavenging capacity; (4) neurotrophic support enhancement via BDNF upregulation; (5) cholinergic pathway modulation through dual effects on ChAT activity and AChE inhibition; and (6) neuroinflammatory response attenuation through balanced MAPK signaling modulation. These observations suggest a multi-pathway approach that warrants further investigation for memory function applications. However, these findings represent preliminary evidence that requires validation through direct molecular binding studies, receptor interaction assays, and mechanistic pathway confirmation. Future in vivo studies and detailed mechanistic characterization are essential to establish causative relationships and advance the therapeutic potential of PBEH for cognitive enhancement disorders.

## Figures and Tables

**Table 1:** Summary of neuroprotective effects of treatments across key pathways in differentiated SH-SY5Y cells.

Pathway	Marker	PBEH	Com-A	Com-B
Aβ aggregation	APP	↓	-	↓
	BACE	↓↓	-	↓↓
	Aβ	↓↓	-	↓↓
Oxidative stress	ROS	↓	–	↓
Synaptic plasticity	BDNF	↑↑	↑↑↑	
	*p*-ERK	↑↑	↑	↑↑
Cell survival	Neuroprotection	↑↑↑	↑↑↑	↑↑
Apoptosis	BAX/BCL-2	↓↓	↓	↓↓↓
	Caspase-3	↓↓	↓	↓↓↓
Cholinergic	ChAT	↑	↑	-
	AChE	↓	-	↓
Inflammation	IL-1β	↓↓	↓	↓↓↓
	*p*-JNK	↓	-	↓
	*p*-p38	↓	-	↓↓

PBEH, porcine brain enzyme hydrolysate; Com-A, N-PEP-12; Com-B, *Ginkgo biloba* leaf water extract; APP, amyloid precursor protein; BACE, β-site amyloid precursor protein cleaving enzyme; Aβ, amyloid beta; ROS, reactive oxygen species; BDNF, brain-derived neurotrophic factor; ERK, extracellular signal-regulated kinase; BAX, BCL2-associated X protein; BCL-2, B-cell lymphoma 2; ChAT, choline acetyltransferase; AChE, acetylcholinesterase; IL-1β, interleukin-1 beta; JNK, c-Jun N-terminal kinase; p38, p38 mitogen-activated protein kinase; p-, phosphorylated. Arrows indicate the direction and magnitude of change: ↑ increase, ↓ decrease; single, double, or triple arrows denote increasing effect size; - indicates no significant change from the control.

## Data Availability

Data is contained within the article or Supplementary Material.

## Supplementary Materials

The following supporting information can be downloaded at https://www.mdpi.com/article/10.3390/ijms26136030/s1.
