# Supplementary material for: Neuroprotective Mechanisms of Porcine Brain Enzyme Hydrolysate in Memory Impairment: Multi-Target Strategy Against Amyloid-β-Induced Neurotoxicity
Source: Int J Mol Sci. 2025 Jun 24;26(13):6030. doi: 10.3390/ijms26136030 (PMC12250036; doi:10.3390/ijms26136030)
Supplement: Supplementary file 1 [file ijms-26-06030-s001.zip › ijms-3680311-supplementary.pdf]

Supplementary figures

Figure S1. Amounts and chromatograms of index compounds

A. Chromatogram of amino acid standards

B. Chromatogram of amino acids

C. Chromatogram of peptide (proline-serine-isoleucine-serine [PSIS]) standards

D. Chromatogram of peptide (PSIS)

Figure S2. Cytotoxicity of porcine brain enzyme hydrolysate (PBEH) in differentiated Sh-SY5Y cells

Differentiated SH-SY5Y cells were treated with PBEH (50–400 μg/mL), and after 24 h, cell survival was measured with a 3-(4,5-dimethylthiazol-2-yl)-2,5-diphenyltetrazolium bromide (MTT) assay.

Figure S1A

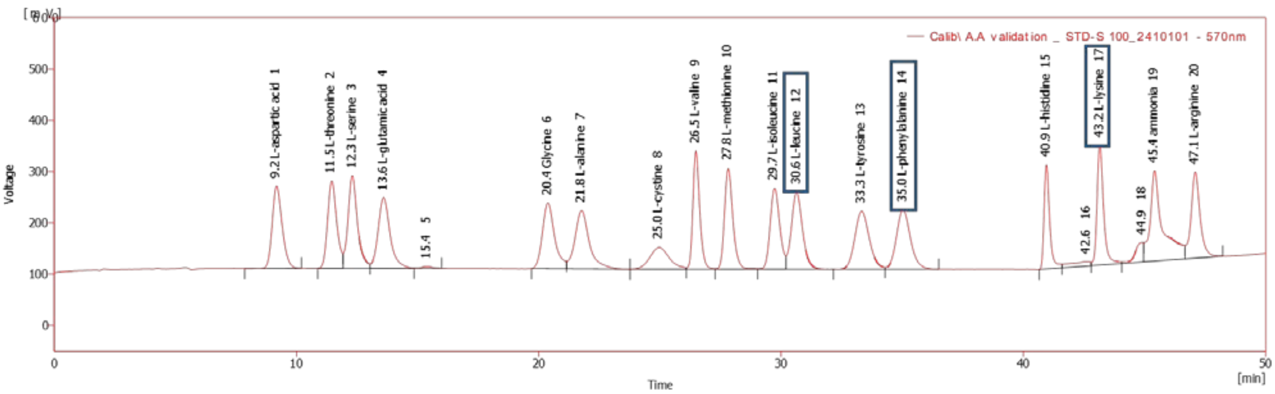

Figure S1B

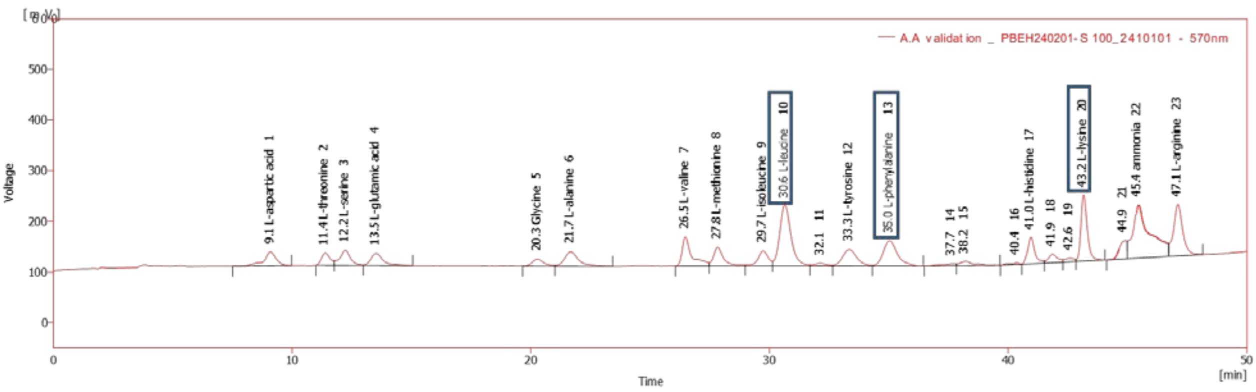

Figure S1C

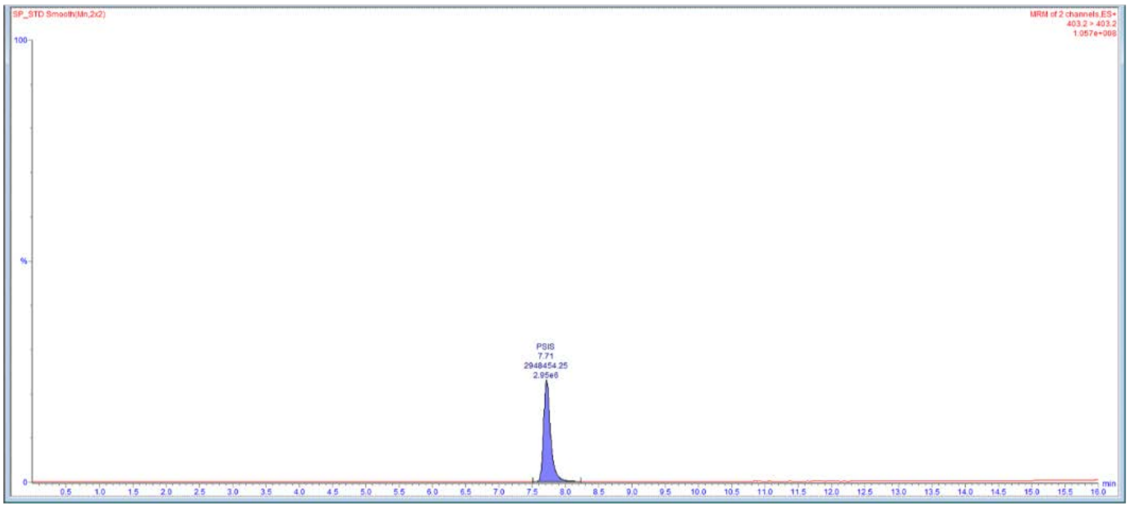

Figure S1D

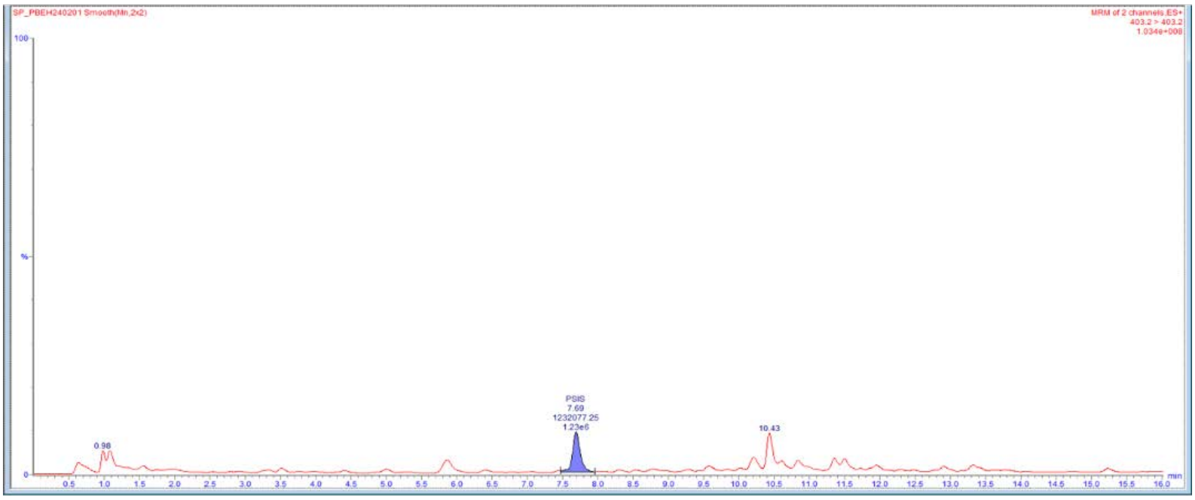

Figure S2

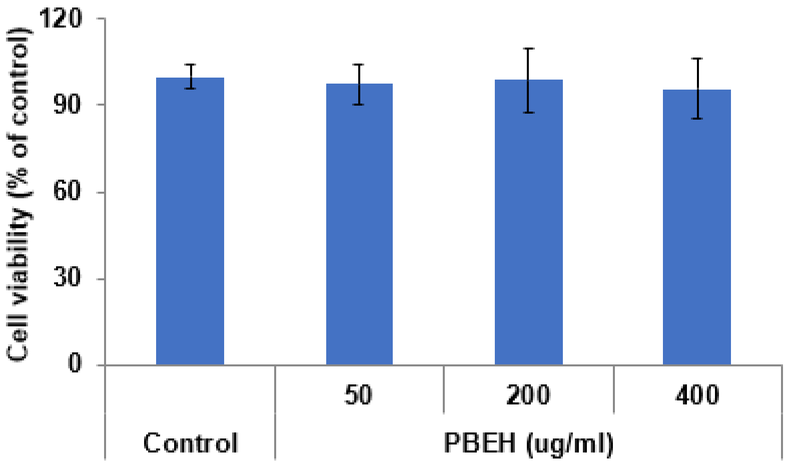

Cell viability (% of control)
120
90
60
30
0
50
200
400
Control
PBEH (ug/ml)
